# S233. EVOLUTION OF THE FUNCIONING AND ATTITUDES TO MEDICATION IN A SAMPLE WITH EARLY PSYCHOSIS IN TREATMENT WITH ARIPIPRAZOLE ONCE-MONTHLY

**DOI:** 10.1093/schbul/sby018.1020

**Published:** 2018-04-01

**Authors:** Marta Soto, Adolfo Benito

**Affiliations:** SESCAM

## Abstract

**Background:**

Patients with first-episode schizophrenia usually respond well to treatment, but relapse is frequent during the first years of the illness and may be associated with clinical deterioration A major concern in treating patients with schizophrenia is non-adherence with medication, with approximately 40% stopping within 1 year and 75% by 2 years. In addition to the impact on the illness, non-adherence creates serious social and psychological consequences. Although it is one of the potentially preventable causes of relapse, hospitalization, and poor outcome non-adherence is one of the most difficult problems to solve

Aripiprazole is an atypical antipsychotic with partial agonist activity at dopamine D2 receptors and a potentially less burdensome metabolic profile compared with other atypical antipsychotics. A once-monthly, long-acting injectable (LAI) formulation, aripiprazole once-monthly 400 mg (AOM 400), is approved for the treatment of schizophrenia

**Methods:**

To assess the evolution of the functioning and attitude to medication in a sample of patients recently diagnosed of schizophrenia during one year of treatment with aripiprazole once-monthly (AoM)

Schizophrenic patients from three Mental Health units in the province of Toledo (Spain) were recruited. The inclusion criteria were an age between 18 and 65 years, a diagnosis of schizophrenia (based on the ICD-10 criteria) in the last 5 years, the start of treatment with AoM in last 6 months, and 12 months of treatment with oral antipsycohtics previosly to the AoM treatment and at least one relapsed in last 4 years. A series of demographic variables were recorded and the GAF (Global Assesment Functioning) scale was used to assess function, while the Drugs Attitude Inventory (DAI) scale was used to evaluate attiudes to medication. The scales were again applied 3, 6 and 12 months after the start of treatment.

**Results:**

N=18 patients (12 males and 6 females), with a mean age of 29 years. There were 1 dropouts during the year of follow-up. The results showed an improvement in GAF score during the 12 months, manifesting from the third month (ANOVA, p<0.05). Likewise, statistically significant differences (ANOVA, p<0.05) were observed with the DAI scale for attitude to treatment; these results persisted over the year of follow-up and were manifest from the third month.

**Discussion:**

First years of evolution in schizophrenia are determinants for the evolution of the illness. The correct adherence to medication is associated with an adecuated functioning

Poor adherence to treatment is a recognised is predictive of poor outcomes (relapse, aggressive behaviour, suicide, substance abuse) and increased risk of hospitalisation Guidelines suggest that long-acting antipsychotic medicines may be offered to people who would prefer such treatment or in cases where avoiding covert non-adherence to antipsychotic medication (either intentional or unintentional) is a clinical priority within the treatment plan.

